# Utilizing Peer Cohort Networks to Disseminate Information and to Prevent and Mitigate COVID-19 in Nursing Homes

**DOI:** 10.1093/geroni/igab046.224

**Published:** 2021-12-17

**Authors:** Rajean Moone

**Affiliations:** University of Minnesota, Woodbury, Minnesota, United States

## Abstract

The COVID-19 pandemic disproportionately impacted residents, families, and staff of nursing homes and senior care communities. Even with federally mandated emergency planning, the pandemic highlighted the lack of preparation to meet the daily challenges faced in senior care. In response, the federal CARES Act including funding for a nationwide network of nursing home cohorts led by academic health centers to disseminate clinical guidance in infection control and pandemic mitigation strategies. We present a case study of a successful diffusion model as implemented in Minnesota with seven cohorts comprised of 242 nursing homes and 544 employees. Experts in geriatric care, long term care regulatory management, and public health led ninety-minute sessions held over the span of sixteen weeks. The session format included foundational evidence-based practices in pandemic management (including infection control, social isolation, leadership, and other topics), individual case studies, peer to peer knowledge diffusion, and expert guidance.

